# Severe abdominal pain and diarrhea – unusual multiple myeloma presentation with a severe prognosis: a case report

**DOI:** 10.1186/s13256-018-1598-y

**Published:** 2018-03-18

**Authors:** Douglas A. Salguero, Pamela A. Barletta, Willaim Sierraalta

**Affiliations:** 1 Advance Simulation Center, University of Francisco Marroquin-UFM, 6 avenue zone 10, 01011 Guatemala, Guatemala; 2 Universidad Latina de Panama, Panama City, Panama; 3 Universidad del Zulia, Maracaibo, Venezuela

**Keywords:** Diarrhea, Abdominal pain, Unusual, Multiple myeloma, Plasmacytoma

## Abstract

**Background:**

Multiple myeloma is a hematologic disease with high mortality rates all over the world. The diagnosis has always been challenging since the first case was reported in 1844. For that reason the diagnostic criteria have evolved over years to include the features of the disease more comprehensively. Unusual presentations are infrequent and a diagnostic challenge. For this reason we report this rare case in which diarrhea and abdominal pain were the initial presenting symptoms of multiple myeloma with a plasmacytoma.

**Case presentation:**

An 87-year-old Hispanic man with a past medical history of hypertension, diabetes, and constipation, presented to an emergency department complaining of severe generalized abdominal pain and profuse diarrhea for 3 days. A physical examination revealed generalized pallor and dehydration but no signs of abdominal peritoneal irritation. Laboratory tests revealed neutrophilia and an elevated total protein. He received intravenously administered fluids and antibiotics. His abdominal pain became localized in the infraumbilical area and a small mass was palpated on the right lower quadrant on subsequent examination. An abdominal computed tomography scan showed a tumor lesion surrounded by fluid collection and a computed tomography-guided biopsy of the lesion confirmed it to be a plasmacytoma. A bone marrow biopsy revealed plasmatic cell augmentation but his beta-2 microglobulin levels were inconclusive. The diagnosis of multiple myeloma was finally confirmed with urine immunofixation. Bortezomib was initiated to decrease disease progression, but unfortunately 4 days later he developed acute pulmonary edema, had a cardiac arrest, and died.

**Conclusions:**

This case illustrates the protean initial manifestations of multiple myeloma and the importance of an accurate diagnosis. Our patient’s initial presentation with gastrointestinal complaints is rare and the plasmacytoma location is even rarer, providing a challenging diagnostic problem. Prompt recognition of multiple myeloma is critical to institute appropriate therapy and prevention of disease progression.

## Background

Multiple myeloma (MM) has a high mortality rate all over the world and it is a challenging disease to diagnose. MM accounts for 1% of all cancers and 15% of all hematologic malignancies. It is more commonly reported in industrialized regions, and relatively little is known about it in Latin America [1]. The initial clinical presentation is commonly fatigue and bone pain with complementary studies revealing anemia, elevated creatinine, hypercalcemia, and osteolytic skeletal lesions.

This plasmatic cell disorder has evolved over the years and now extramedullary involvement and the grade of the bone marrow affected are taken into account; following the current guidelines provided by the International Myeloma Working Group (IMWG), a plasmacytoma with more than 10% of bone marrow affected is indicative of MM. The most frequent plasmacytoma location (accounting for 49%) is the axial skeleton. In this case, we report a rare appendicular plasmacytoma in the abdomen with 31% of plasma cell proliferation in bone marrow.

An 87-year-old Hispanic man with a past medical history of hypertension, diabetes, and chronic constipation, presented to an emergency department complaining of severe generalized abdominal pain and profuse diarrhea for 3 days. A physical examination revealed dehydration and a localized abdominal pain in the infraumbilical area with a small mass on the right lower quadrant. An initial laboratory work was benign, only revealing a decreased albumin/globulin ratio, and neutrophilia, all other laboratory findings were under normal values. A biopsy of the mass revealed a plasmacytoma that prompted the workup, revealing a urine immunofixation for IgG gammopathy. He died 17 days after the initial presentation from an intractable pulmonary edema and cardiopulmonary arrest (Table 1).

**Table 1:** Relevant past medical history and interventions

Date	Summaries from initial and follow-up visits	Diagnostic testing	Interventions
Day 1	Patient’s primary concerns were 3 days of diarrhea and generalized abdominal pain	• Abdominal X-rays: generalized colon distension without obstruction signs • Chest X-rays: pleural fluid in the left basal lung • Chest CT scan: atelectasis in the left basal lung • Neutrophilia • Decreased albumin/globulin ratio • Normal electrolytes • Normal serum osmolality • Normal stool test	In the emergency room: • endogenous fluids • antibiotics • albumin
Day 2	We found a right lower quadrant mass on abdominal palpation. Patient showed significant clinical improvement	• Abdominal CT with contrast: sigmoid diverticulosis and a 4.96 cm diameter right iliac crest mass • Biopsy of the mass: plasmacytoma • Thorax CT: severe lytic lesions • Skeletal survey: multiple lytic lesions in the skull, femur, and humeral head • Hyperproteinuria • Alpha-2 monoclonal peak	• He was tolerating oral daily diet • Diarrhea stopped • Physical therapy • Pulmonary-respiratory therapy with vibration and positive pressure exercises
Day 5	Clinically stable	• Urine immunofixation came back positive for monoclonal gammopathy component type Kappa IgG • Bone marrow biopsy: high expression of CD38 and CD56, compatible with a plasmatic cell neoplasia	• Multiple myeloma was confirmed • Bortezomib was started
Day 12	Acute productive cough of white sputum	• Thorax X-rays: pulmonary edema plus new costal fractures • BNP was elevated to 307 pg/ml • Hb was decreased to 10 mg/dl	• Transferred to intensive care unit • Furosemide • Erythropoietin • Transfused with pack red blood cells
Day 17	Mental status progressively deteriorated. Cardiorespiratory arrest	• BNP increased to 617.9 pg/ml • Thorax X-rays: increased cardiac silhouette	• Do not resuscitate agreement with the family

An 87-year-old Hispanic man with a family history including hypertension. Past medical history of hypertension, type 2 diabetes mellitus, and chronic constipation controlled with valsartan/amlodipine/insulin glargine. Non-tobacco smoker; occasionally drinks alcohol. *BNP* brain natriuretic peptide, *CT* computed tomography, *Hb* hemoglobin

We report this case with the purpose of describing an unusual clinical and uncommon laboratory manifestation of MM; the diagnosis was very challenging and you should be aware and take care when you find an elderly patient presenting with acute severe abdominal pain, diarrhea, and malignancy risk factors, especially because of the high mortality rate.

## Case presentation

An 87-year-old Hispanic man presented to an emergency room complaining of a 3-day history of abdominal pain and profuse diarrhea. He stated that initially he was feeling constipated, which is the reason he was prescribed with over the counter enemas. Four days later he started to feel generalized abdominal colic-type pain that progressively increased until it became unbearable; he also experienced fever and watery stools for a day, which prompted him to seek care in the emergency room. He denied having bone pains, weight loss, or fever. He had a past medical history of hypertension, diabetes, and chronic constipation and was taking insulin glargine, amlodipine, and valsartan. He had a past family history of hypertension and denied having allergies, surgical history, or trauma history. He stated that he was a lifelong non-smoker of tobacco and consumed alcohol occasionally.

An initial physical examination showed a man in mild distress and with dry mucous membranes. His vital signs were normal except for a blood pressure of 150/87 mmHg. His abdomen was non-distended but tender, especially on deep palpation, with no rebound tenderness. No masses or signs of peritoneal irritation were noted, bowel sounds were normal, and a rectal examination showed a normal sphincter tone, no masses, and no signs of rectal bleeding.

**Fig. 1:**
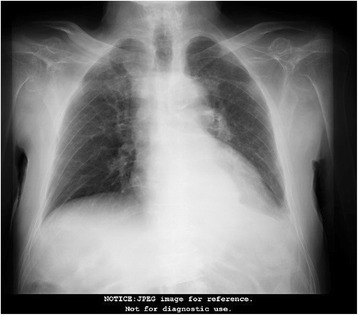
Chest X-ray on admission. Pleural fluid in the left basal lung area and dorsal vertebral column with signs of osteopenia

Laboratory testing revealed hemoglobin 13.8 g/dl, hematocrit 39%, white blood cell (WBC) count 6.1 K/uL, neutrophils 71.3%, platelets 197 K/uL, creatinine 1.06 mg/dl, blood urea nitrogen (BUN) 30.2 mg/dl, serum total protein 11.6 g/dl, albumin/globulin ratio 0.2 (normal > 1), globulin 9.7 g/dl (2.3–3.5), calcium 8.4 mg/dl (8.4–10.2), and phosphate 4.0 mg/dL. A viral stool panel was negative. Stool for occult blood and *Clostridium difficile* toxin were both negative. A chest X-ray (Fig. 1) showed a left-sided pleural effusion and osteopenia in the thoracic spine. A supine abdominal X-ray denoted generalized colonic distention without signs of bowel obstruction.

**Fig. 2:**
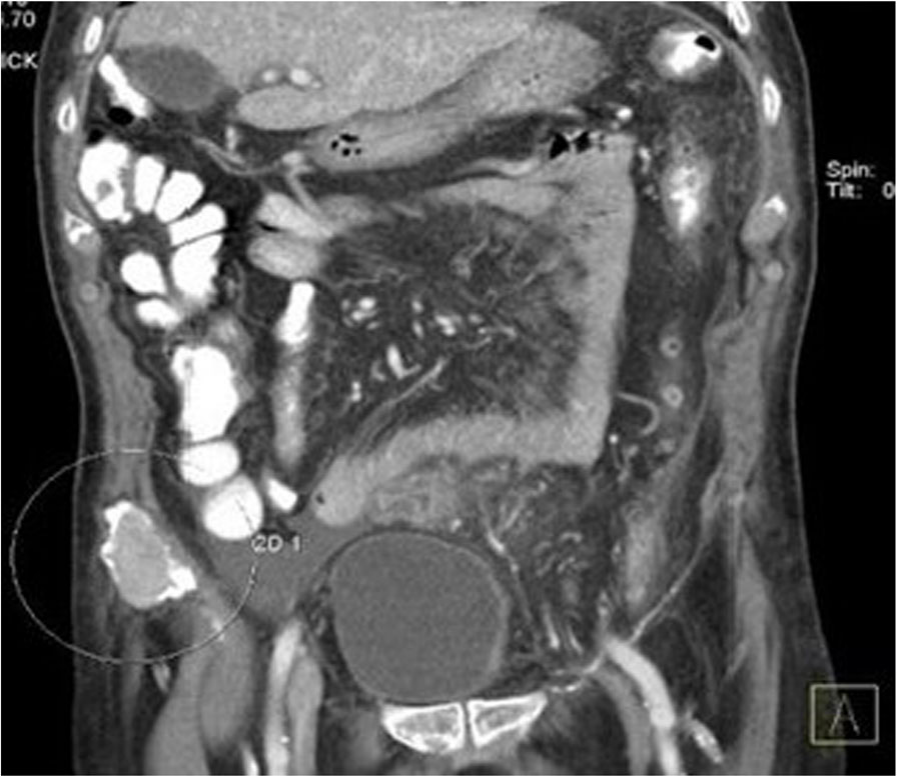
Computed tomography scan with contrast of the abdomen. Sigmoid diverticulosis and a 4.96 cm diameter right iliac crest mass, accompanied by 6.5 × 4.8 cm of fluid collection. The circle is showing the right iliac crest mass that was confirmed as a plasmacytoma

**Fig. 3:**
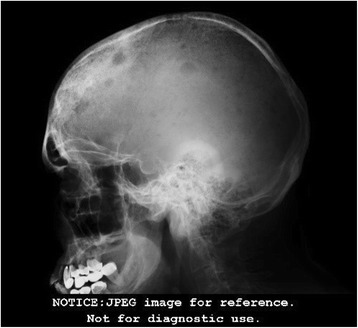
Lateral X-ray of the head. Multiple lytic lesions in the skull

**Fig. 4:**
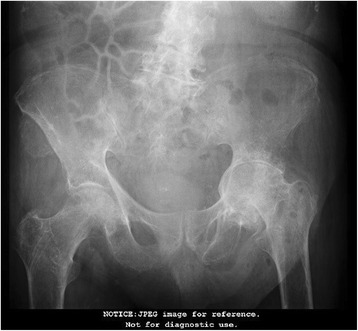
Anteroposterior X-ray of the pelvis. Multiple lytic lesions in the femur and humeral head

**Fig. 5:**
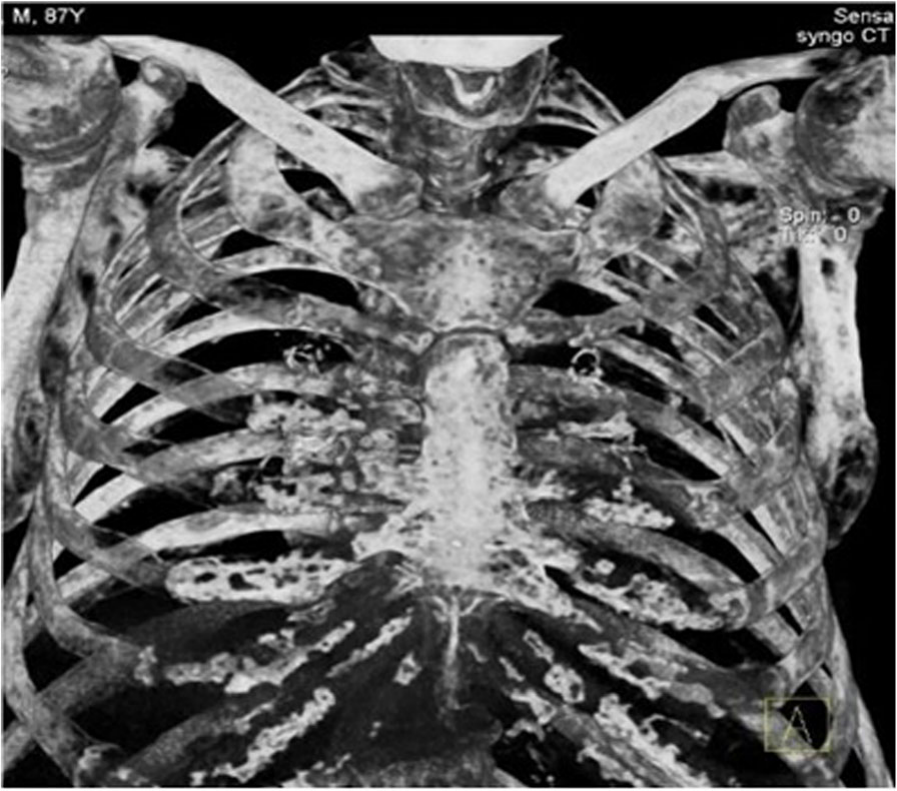
Chest computed tomography scan. Severe lytic lesions involving several costal ribs

Based on these results, our patient was treated with metronidazole, ceftriaxone, and albumin infusions; he was reevaluated and found to have a right lower quadrant mass on abdominal palpation. An abdominal computed tomography (CT) scan with contrast (Fig. 2) showed a 4.96 cm right iliac crest mass. The mass was biopsied and showed findings compatible with a plasmacytoma. This prompted further testing. His urine protein was 1046.25 mg/24 hours (50–150), beta-2 microglobulin 3.7 mg/L (0.7–3.4 mg/L), and a skeletal X-ray survey showed multiple lytic lesions in his skull, femur, and humeral head (Figs. 3 and 4), and a thorax CT revealed severe lytic lesions involving several costal ribs (Fig. 5). Protein electrophoresis showed an alpha-2 monoclonal peak.

Two days after starting antibiotics our patient had a significant improvement; he was tolerating oral daily diet, his abdominal pain had decreased, and his diarrhea stopped. Urine immunofixation revealed a monoclonal Kappa IgG gammopathy and a bone marrow biopsy showed a high expression of CD38 and CD56, compatible with a plasmatic cell neoplasia. He started chemotherapy with bortezomib.

**Fig. 6:**
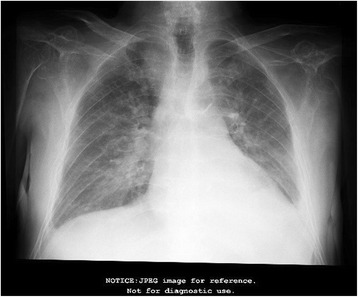
Chest X-ray on day 12. Pulmonary edema plus new costal fractures

**Fig. 7:**
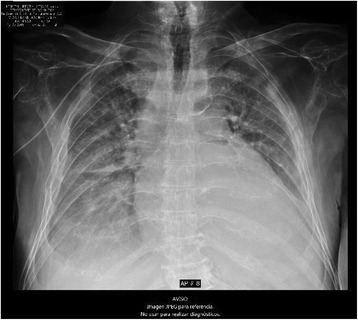
Chest X-ray on day 17. Increased cardiac silhouette

On day 12, he developed a productive cough with white sputum. X-rays showed a pattern compatible with pulmonary edema and new costal fractures (Fig. 6). His brain natriuretic peptide (BNP) was elevated at 307 pg/ml (< 100 pg/ml) so furosemide was administered. He was transferred to the intensive care unit (ICU), where his mental status progressively deteriorated until he remained somnolent and lethargic. His BNP increased further to 617.9 pg/ml (< 100 pg/ml) and a new chest X-ray showed an enlarged cardiac silhouette (Fig. 7). On his 17th day of hospitalization he developed cardiopulmonary arrest and died.

### Outcome and follow-up

The disease progression could not be stopped with the first-line treatment and support therapy, resulting in the death of our patient in less than a month.

## Discussion

We report a case of an elderly Hispanic man with an unusual clinical and laboratory manifestation of MM. We could not ascribe an etiology for his abdominal pain and diarrhea; it was the presence of a decreased albumin/globulin ratio and a mass confirmed later to be a plasmacytoma that prompted the workup for the final diagnosis. The initial therapy was started as soon as the diagnosis was confirmed but despite chemotherapy he developed intractable pulmonary edema which caused his death.

MM is a systemic plasma cell disorder accounting for 1.5% of all cancers [2]. Solitary plasmacytoma (SP) is a rare independent subset of plasma cell disorders characterized by a localized proliferation of neoplastic monoclonal cells. It has a very low incidence and, given its rarity, very limited information is available [3]. Both conditions have notable similarities in terms of their cytological and immunophenotypic characteristics; however, these tumors are differentiated by their site predilection and mortality rates [3]. Our patient had MM with plasmacytoma with bone marrow involvement of 31%, with high expression of CD38 and CD56, and multiple bone lytic lesions on X-rays confirmed with a CT scan.

The current guidelines provided by the IMWG (2013) define SP as biopsy-confirmed plasma cell proliferation in the bone or soft tissue in the presence of normal bone marrow and normal skeletal survey. Guidelines also recommend using the term SP when less than 10% of bone marrow is involved without osteolytic lesions. A plasmacytoma with more than 10% of plasma cell proliferation in bone marrow has always been indicative of MM [4]. With this in mind, we classified our patient as having MM. The major difficulty in MM is the disease definition because it is clinicopathological; it needs overt clinical manifestations of serious end organ damage, such as osteolytic bone lesions and renal failure, before the diagnosis can be made. We found a rare appendicular plasmacytoma in our patient; his symptoms varied at the initial presentation. This is often deleterious for patients because they receive treatment at an advanced stage of the disease; however, current treatment options have greatly improved, showing that prompt treatment of a high risk initial presentation of a patient with plasmatic cell neoplasia can extend survival rates [5, 6].

SP is an infrequent plasmatic cell neoplasia, with only 1691 cases reported in the last 9 years in the USA by the Surveillance, Epidemiology, and End Results program (SEER) search [3, 7]. In comparison, MM affects nearly 20,000 patients each year [8]. Given the rarity of this neoplasia it is important to mention some important facts related to our patient. Out of all patients with SP, 80% are white men over 60-years old and it is rare in other ethnic groups (only 5.9%), bone being the most common site of involvement in 57.78% of cases; the axial skeleton is the most frequent bone structure involved (49.14%) while the appendicular skeleton (iliac crest and extremities) is the rarest (only 8.63%) [3]. Patients with SP involving the appendicular skeleton have the most unfavorable outcomes [3, 9]. Most frequently, a plasmacytoma diagnosis was made before MM, which is a progression of the initial plasmacytoma [3].

It is important to note that the most common presenting symptoms of MM are fatigue and bone pain [4, 10]. Anemia occurs in approximately 75% of patients, and osteolytic skeletal lesions can be detected in approximately 80% of patients [10, 11]. Common laboratory findings at presentation include hypercalcemia (15%) and elevated creatinine > 2 mg/dl (20%) [11]. Extramedullary involvement occurs in only 1 to 2% of patients with MM at the time of diagnosis, while an additional 8% develop extramedullary disease later in the disease course [11].

Most patients present with symptoms related to their initial presentation. In a large study, bone pain/discomfort was the most common symptom reported in MM with 73.7% presenting with a disease-specific symptom [10]. In contrast, from 108 patients presenting with abdominal pain, only eight were diagnosed as having MM, excluding abdominal pain as a frequent disease-specific symptom for MM.

Furthermore, an excessive diagnosis delay (> 3 months) may result in end organ damage such as renal failure and fracture, contrasting with our patient who presented with acute symptoms and severe disease complications in a short period [10]. Patients who did not think their symptoms were serious were more likely to seek a specialist after a period of more than 3 months; among these symptoms abdominal pain/discomfort was one [10, 12]. Rapid diagnosis may result in favorable patient outcomes, including fewer complications and reduced mortality [5, 10, 11]. We spent less than 1 week in determining the correct diagnosis of our patient and 2 more weeks in treatment, however, despite all the effort unfortunately he died from a cardiac arrest.

Our patient manifested a rapid disease progression and a poor prognosis. During an early stage of chemotherapy, he manifested physical signs of congestive heart failure (CHF) and an electrocardiogram (EKG) revealed atrial fibrillation. His baseline echocardiogram showed a non-compromised ejection fraction of 58%, moderate pulmonary hypertension of 48 mmHg, aortic stenosis, and left atrial enlargement. We could not relate these findings with his past medical history, which was only hypertension and diabetes, both controlled, and he never mentioned these kinds of symptoms.

As we know, MM complications such as amyloid light-chain (AL) amyloidosis can cause a cytotoxic effect in blood vessels [13]. By deposition, AL amyloidosis can even impair cardiac valves, it can impair the conduction system increasing the incidence of arrhythmias (atrial fibrillation is the most common affecting 10–15% of patients), and it is associated with significant morbidity related to heart failure [13, 14]. In addition, with MM complications, the cardiovascular (CV) toxicity spectrum of bortezomib was reported in recent studies and case reports revealed an increased risk of CV events [14]. Seen approximately in 15% of patients with bortezomib treatment, the most commonly reported cardiac adverse events were decreased left ventricular ejection fraction, CHF, arrhythmias, and ischemic heart disease [14], due to proteotoxicity and apoptosis of cardiomyocytes [14].

### Learning points

 •MM has several types of presentation and for this reason it is important to clarify unusual types of manifestation in the elderly population, such as abdominal pain.

•Early diagnosis of MM or plasmacytoma is very important due to its high mortality rates; this is why we should include it in our differential diagnosis of abdominal pain in elderly populations.

•A high clinical suspicion must be present during the evaluation of an elderly patient with a normal initial blood work with malignancy risk factors.

•MM diagnosis criteria have been modified since 2014 to achieve higher accuracy and simplify the method.

•Bad prognosis signs should be clear during the diagnosis, selection of chemotherapy, and management of disease during hospitalization.

•MM complications have high mortality rates.

## Conclusions

In current times, MM has been categorized as one of the most common hematologic diseases. In the past, this disease was barely diagnosed until severe symptoms occurred, now we can diagnose and treat the patients early, giving a better prognosis. Very few studies have been documented with diverse types of disease manifestation at initial presentation; this is why we report this case because an unusual presentation is important to keep in mind, especially abdominal pain in elderly patients, due to its very high mortality rate. No studies have been conducted evaluating a relationship between initial presentation of SP or MM and mortality prognosis. For this reason, this case goes beyond our knowledge of how we can increase the survival rate in these kind of patients; if an early diagnosis is enough rather than just chemotherapy combination, or if a different approach can be given to these patients. In conclusion, this could be a good starting point for future studies.
